# A Sub-mW 18-MHz MEMS Oscillator Based on a 98-dBΩ Adjustable Bandwidth Transimpedance Amplifier and a Lamé-Mode Resonator

**DOI:** 10.3390/s19122680

**Published:** 2019-06-13

**Authors:** Anoir Bouchami, Mohannad Y. Elsayed, Frederic Nabki

**Affiliations:** Department of Electrical Engineering, École de technologie supérieure, Montréal, QC H3C 1K3, Canada; bouchami.anoir@courrier.uqam.ca (A.B.); mohannad.elsayed@mail.mcgill.ca (M.Y.E.)

**Keywords:** MEMS-based oscillator, transimpedance amplifier, electrostatic actuation, phase noise, Lamé-mode MEMS resonator, input-referred noise, quality factor, 1-dB compression point

## Abstract

This paper presents a microelectromechanical system (MEMS)-based oscillator based on a Lamé-mode capacitive micromachined resonator and a fully differential high-gain transimpedance amplifier (TIA). The proposed TIA is designed using TSMC 65 nm CMOS technology and consumes only 0.9 mA from a 1-V supply. The measured mid-band transimpedance gain is 98 dBΩ and the TIA features an adjustable bandwidth with a maximum bandwidth of 142 MHz for a parasitic capacitance $C_{P}$ of 4 pF. The measured input-referred current noise of the TIA at mid-band is below 15 pA/$\sqrt{\mathrm{Hz}}$. The TIA is connected to a Lamé-mode resonator, and the oscillator performance in terms of phase noise and frequency stability is presented. The measured phase noise under vacuum is −120 dBc/Hz at a 1-kHz offset, while the phase noise floor reaches −127 dBc/Hz. The measured short-term stability of the MEMS-based oscillator is ±0.25 ppm.

## 1. Introduction

Oscillators are of great interest because of their ubiquitous use in timing applications and in modern wireless communication devices. They are indispensable to ensure proper synchronization in almost any system. Micromachined resonators, a subset of microelectromechanical systems (MEMS), are receiving continuously increasing interest due to their small sizes as well as their potential for integration with other integrated devices and circuits on the same chip which makes them excellent candidates for replacing crystal-based resonators in timing applications. This is especially important for handheld and wearable electronic applications where weight, size, and cost are critical parameters. However, resonator performance, mainly operation frequency ($f_{0}$), quality factor (Q), resonator parasitics and motional resistance, set stringent requirements for the oscillation sustaining circuitry (e.g., gain and power consumption) in order to achieve a high-performance MEMS oscillator.

Resonators can be classified based on their vibration modes as either flexural or bulk-mode devices. Bulk-mode devices typically exhibit high stiffness, and are consequently less prone to thermoelastic damping, compared to flexural devices, allowing them to achieve large quality factors (>10,000), even at atmospheric pressure [1,2,3,4,5,6,7,8,9,10,11,12,13,14,15,16]. In [2,3,4,5,6,7], bulk-mode resonators including Lamé-mode and wine glass mode devices with quality factors in the 10${}^{6}$ range were presented based on capacitive actuation. Such high quality factors are achievable as a result of the superior structural material, single crystalline silicon, which is patterned into a pure bulk resonating structure, without the need for release holes, or additional layers on top, which is one of the limitations for the quality factors achieved by piezoelectrically actuated bulk-mode devices (e.g., [8,9,10]). On the other hand, capacitive bulk-mode devices typically exhibit lower transduction efficiencies compared to piezoelectric devices, which translate to higher losses and motional resistances. This can be accounted for by either enhancing the transduction, e.g., sub-micron gaps realized by complex fabrication processes [11,12,13], high voltages [1,2,3,4,5,6,7,14], added transducer combs [15,16], movable electrodes for gap closing [17,18], or increasing the gain for the transimpedance amplifier (TIA) in order to sustain oscillation. Several transimpedance topologies have been reported in the literature for MEMS-based oscillator applications [19,20,21,22,23,24,25,26]. Designs proposed in [19,20,21] use an automatic gain control circuit to regulate the oscillation amplitude and reduce the resonator mechanical non-linearity effect. Furthermore, while differential TIAs allow for better performance, the power consumption of fully differential transimpedance amplifier designs in [19,22] are higher than single-ended TIAs in [20,21,23,24,25].

Typically, a MEMS oscillator is realized by connecting a TIA with the resonator in a positive feedback loop to sustain a steady-state oscillation by converting the resonator output current to an output voltage signal and providing sufficient gain and suitable phase. The quality of the output oscillation is usually determined by the quality factor of the resonator and by the electrical noise of the TIA. It is necessary for the TIA to have high transimpedance gain due to the insertion loss of the resonator caused by its motional resistance. A sufficiently wide bandwidth is also required to ensure that the oscillator phase shift is around 0°, when the MEMS-based oscillator operates in series resonance. Thus, Barkhausen conditions will be fulfilled [27]. Furthermore, low input and output impedances are required to minimize the resonator Q-factor loading.

Electrostatic MEMS resonators typically exhibit relatively high loss which necessitates the use of a high-gain transimpedance amplifier in the feedback path to compensate for the losses. While an inverting amplifier can be used (i.e., Pierce oscillator structure), if the circuit bandwidth can be made sufficiently large, the oscillation frequency can be accurately generated with a non-inverting amplifier, without relying on external capacitors and their values in order to ensure the 180° additional phase shift required [28]. This also yields a more compact system, not requiring these off-chip capacitors.

This work proposes a MEMS oscillator that is based on the Lamé-mode resonator presented in [1]. The oscillator includes a fully differential high-gain TIA that is tailored to the resonator. Accordingly, the oscillator achieves very competitive performance in terms of power consumption and phase noise. The paper begins with a summary description of the Lamé-mode resonator followed by detailed descriptions of each of the TIA’s building blocks. Measurement results are then presented and discussed, and are followed by a conclusion.

## 2. Lamé-Mode MEMS Resonator Overview

**Figure 1:**
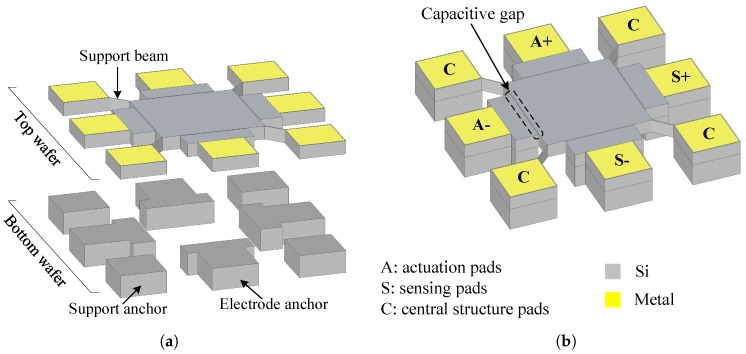
Simplified diagram of the (**a**) exploded and (**b**) assembled views of the Lamé-mode MEMS resonator with corner supports [1].

**Figure 2:**
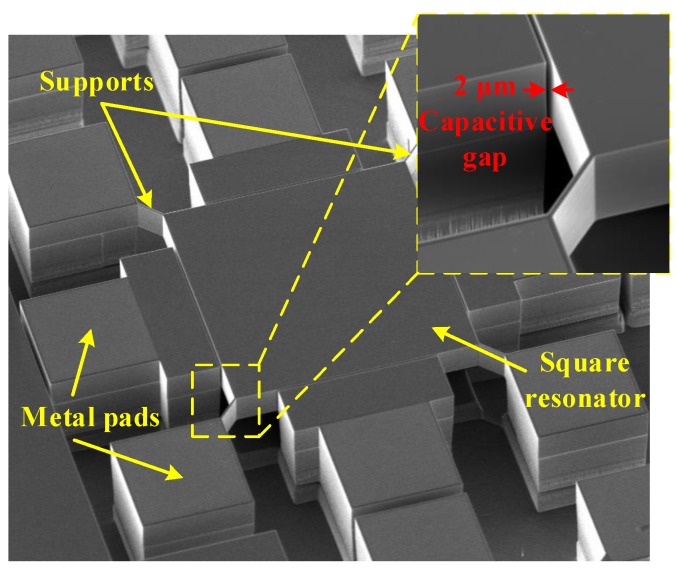
SEM micrograph of the Lamé-mode MEMS resonator with corner supports [1].

A brief description of the Lamé-mode capacitive (i.e., electrostatic) MEMS resonator presented in [1] is given in this section. Figure 1 illustrates exploded and assembled 3D renditions of the resonator structure. Structures were fabricated in a commercial silicon-on-insulator (SOI) technology, MicraGEM-Si, where they are realized through processing and wafer bonding of two SOI wafers (i.e., the top wafer and the bottom wafer). The top wafer has its handle layer removed after bonding to the bottom wafer such that the resonator is mainly composed of a single crystalline silicon central square suspended structure acting as the Lamé bulk-mode resonator. This suspended square is 30 μm thick, has a 230 μm side length, and is formed in the device layer of the top SOI wafer. The resonator square structure is anchored to the substrate through four suspension beams placed at the corner nodal points of the resonance mode. Pads for an electrical connection to the central square are present at the end of each suspension beam. This allows the connection of the DC polarization voltage required for the electrostatic actuation of the device. These support beams are patterned in the device layer of the top SOI wafer. The central structure is surrounded by four electrodes used for capacitive actuation and sensing of the structure. The electrodes are formed in the device layer of the top SOI wafer and are separated from the central square by a 2 μm capacitive transduction gap, which is the minimum spacing allowed by the technology. The device layer of the bottom SOI wafer is patterned to form the electrode anchors and the anchors at the end of the suspension beams. SEM micrographs of the resonator are shown in Figure 2. FEM simulations as well as theoretical calculations predict a resonance frequency of 17.9 MHz.

## 3. Transimpedance Amplifier Circuit Design

**Figure 3:**
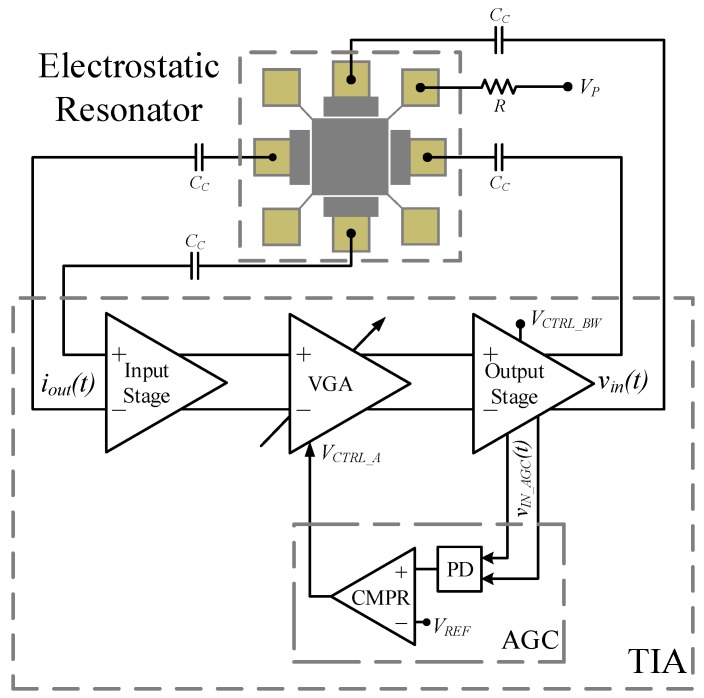
MEMS-based oscillator functional diagram.

**Figure 4:**
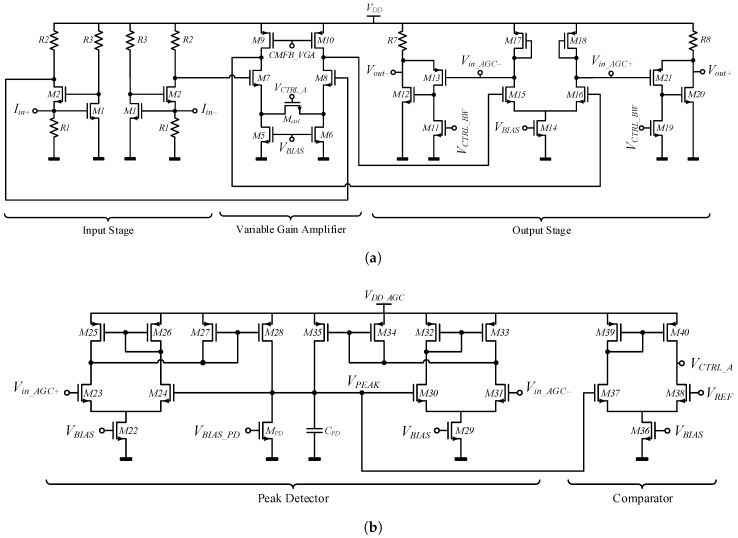
Circuit schematic of: (**a**) the proposed fully differential TIA design, and (**b**) the AGC.

The TIA circuit shown in Figure 3 is composed of a three fully differential stages: (i) an input stage followed by (ii) a variable gain amplifier (VGA) controlled by an automatic gain control circuit (AGC), and (iii) an output stage implemented with a super source follower (SSF). The complete schematic circuit is shown in Figure 4 in which the biasing and common-mode feedback (CMFB) circuits are not shown. The sustaining amplifier provides low input impedance ($R_{in}$) and low output impedance ($R_{out}$) so as to compensate for large interconnect parasitic capacitance ($C_{P}$ = 4 pF) and push the dominant pole far beyond the oscillation frequency. This translates into a high-gain-bandwidth (GBW) product [29].

The gain of the TIA needs to be high enough to compensate for the motional resistance of the MEMS resonator and sustain the oscillation. The regulated cascade (RGC) topology [30] was chosen as the input stage to achieve a reasonable trade-off between gain, bandwidth, and power consumption. The input impedance of the RGC input stage is given by

(1) $$R_{in}=\frac{1}{g_{m2}\;\left(1+R_{3}\;g_{m1}\right)},$$

where $g_{m1}$ and $g_{m2}$ are the transconductances of transistors M1 and M2, respectively. Thus, a smaller input impedance can be attained by increasing the voltage gain of the local feedback stage given by $\left(1+R_{3}\;g_{m1}\right)$. The gain of the input stage is given by

(2) $$Z_{T}\left(s\right)≅\frac{R_{2}\left(1+s\frac{R_{3}C_{1}}{1+R_{3}g_{m1}}\right)}{\left(1+sR_{1}C_{in}\right)\left(1+sR_{3}C_{1}\right)\left(1+sR_{2}C_{gd2}\right)},$$

where $C_{in}$, $C_{1}$, and $C_{gd2}$ are the total input capacitances of the input stage, the equivalent capacitance between the drain of M1 and gate of M2, and the gate-drain capacitance of transistors M2, respectively. To achieve a higher gain, $R_{2}$ should be increased, although it cannot be arbitrarily enlarged because of design constraints. It can be seen from Equation (2) that the 3-dB bandwidth of the input stage is limited by the dominant pole appearing at the drain of transistor M1 and is given by

(3) $$f_{-3dB}=\frac{1}{2\pi R_{1}C_{in}}≅\frac{1}{2\pi R_{1}\times \left(C_{gs1}+C_{gd1}R_{3}g_{m1}\right)},$$

where $C_{gd1}$ and $C_{gs1}$ are the gate-drain capacitance and the gate-source capacitance of transistor M1, respectively. The local feedback of the input stage generates a zero at a frequency of

(4) $$f_{z}≅\frac{g_{m1}}{2\pi C_{1}}≅\frac{g_{m1}}{2\pi \left[C_{gd1}+C_{gd2}\left(1+\frac{R_{2}}{R_{1}}\right)\right]}.$$

To maintain the zero far away from the dominant pole [31], the gate-drain capacitance of transistor M2 should be reduced by decreasing its width. In this fashion, the RGC input impedance in Equation (1) will not be dramatically affected since $g_{m2}$ will not decrease considerably as it is proportional to $\sqrt{{\left(W/L\right)}_{2}}$, while its gate capacitance is linearly proportional to ${\left(WL\right)}_{2}$. This can be compensated by increasing $R_{3}$ as the input impedance is inversely proportional to $\left(1+R_{3}\;g_{m1}\right)$, as shown in Equation (1).

**Figure 5:**
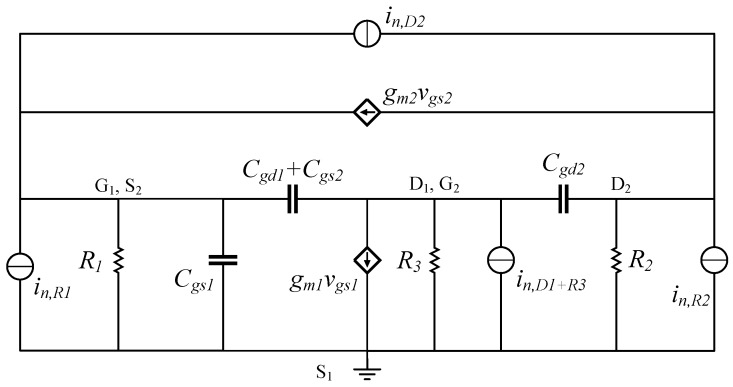
Simplified equivalent circuit of the RGC input stage used for noise analysis.

The input-referred current noise is a key performance parameter to be considered when designing the proposed TIA. It can be used to provide a representative comparison between different circuit topologies. Since the noise is mostly contributed by the input stage, the noise of the other stages can be neglected. Therefore, a noise analysis is carried-out using the equivalent circuit shown in Figure 5, and is based on the analysis method proposed in [31], where shot noise and flicker noise are neglected. Assuming that all the noise sources are uncorrelated, the input-referred current noise of the input stage can be shown to be given by

(5) $$\begin{array}{ccc} \bar{i_{n,in}^{2}} & =\frac{4kT}{R_{1}}+\frac{\omega^{2}{\left(C_{1}+C_{2}\right)}^{2}}{g_{m2}^{2}}\left(\gamma g_{d0,2}+\frac{1}{R_{2}}\right) & +\frac{4kT\left(\frac{1}{R_{1}^{2}}+\omega^{2}C_{in}^{2}\right)}{{\left(g_{m1}+\frac{1}{R_{3}}\right)}^{2}}\left(\gamma g_{d0,1}+\frac{1}{R_{3}}\right), \end{array}$$

where γ is the noise coefficient [32,33], *k* is Boltzmann’s constant, *T* is the absolute temperature, and $g_{d0,1}$ and $g_{d0,2}$ are the zero-bias drain conductance of transistors M1 and M2, respectively. From (5), the noise can be analyzed as follows: the thermal noise contribution from $R_{1}$ is directly referred to the input, and as the frequency increases, the noise is dominated by terms containing $\omega^{2}$. Therefore, a low input-referred noise can be achieved by increasing resistor $R_{1}$, yielding better TIA noise performance.

## 4. Experimental Results

**Figure 6:**
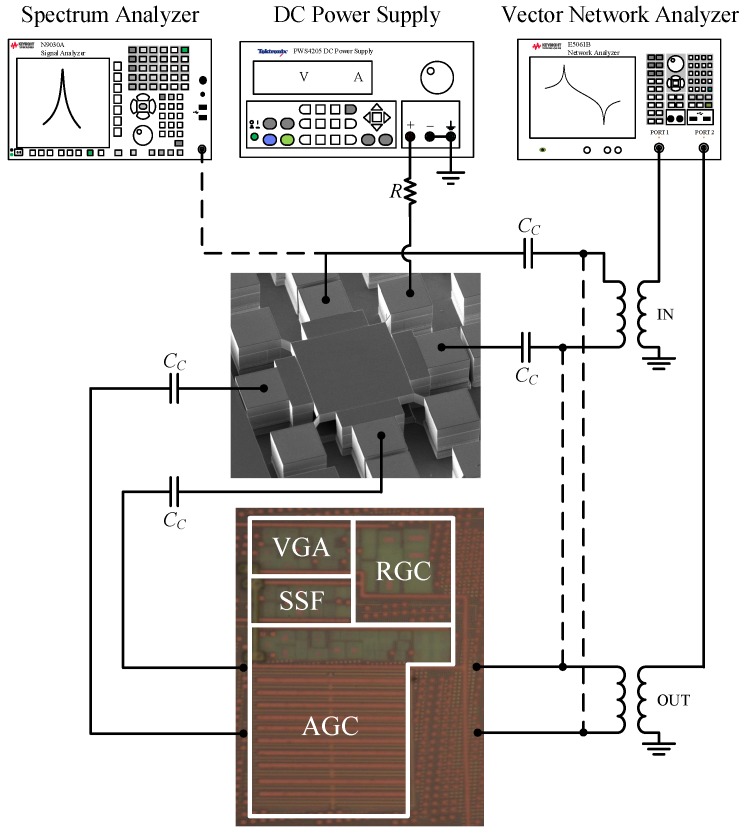
Test setup of the MEMS-based oscillator in open-loop (solid lines) and closed-loop (dashed lines) with micrographs of the TIA and resonator.

The resonator and the TIA were both characterized, and were then combined to implement the MEMS-based oscillator. Two test configuration setups shown in Figure 6 were used to characterize the MEMS-based oscillator: (i) the open-loop configuration and (ii) the closed-loop configuration.

### 4.1. Resonator Characterization

**Figure 7:**
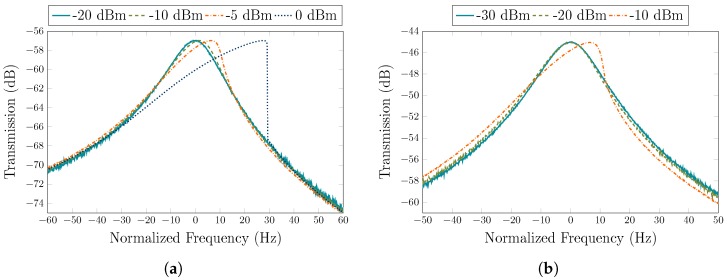
Normalized resonator transmission characteristic curves for various output input amplitude levels for (**a**) V${}_{p}$ = 100 V and (**b**) V${}_{p}$ = 200 V.

The frequency response of the resonator was measured in differential configuration with the VNA under a vacuum level of 100 mTorr for DC polarization voltages, $V_{p}$, of 100 V and 200 V, and for various input power levels starting from −30 dBm up to 0 dBm. Figure 7 shows the transmission characteristic curves normalized to the center frequency of 17.93 MHz. The resonator exhibits a Q-factor of ∼890,000, and a peak transmissions of −57 dB and −45 dB for $V_{p}$ = 100 V and $V_{p}$ = 200 V, which correspond to motional resistances of 35 kΩ and 8.8 kΩ, respectively. The results at high input power levels indicate spring-hardening non-linear behavior, as the Lamé-mode resonator geometry is aligned with the <100> crystalline silicon orientation [34,35,36]. Therefore, a positive amplitude–frequency (*A*–*f*) coefficient (κ) is associated with this resonator [37].

### 4.2. Transimpedance Amplifier Characterization

**Figure 8:**
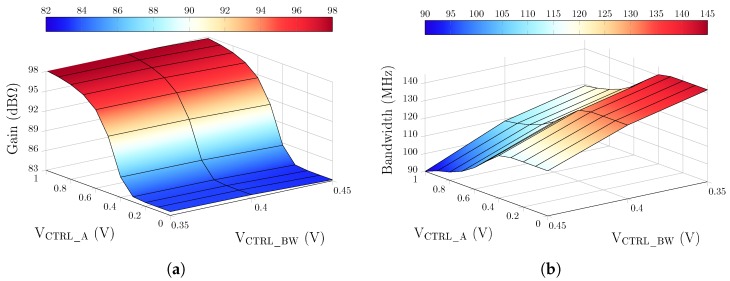
Measured TIA (**a**) gain and (**b**) bandwidth, for different values of $V_{CTRL\_A}$ and $V_{CTRL\_BW}$.

**Figure 9:**
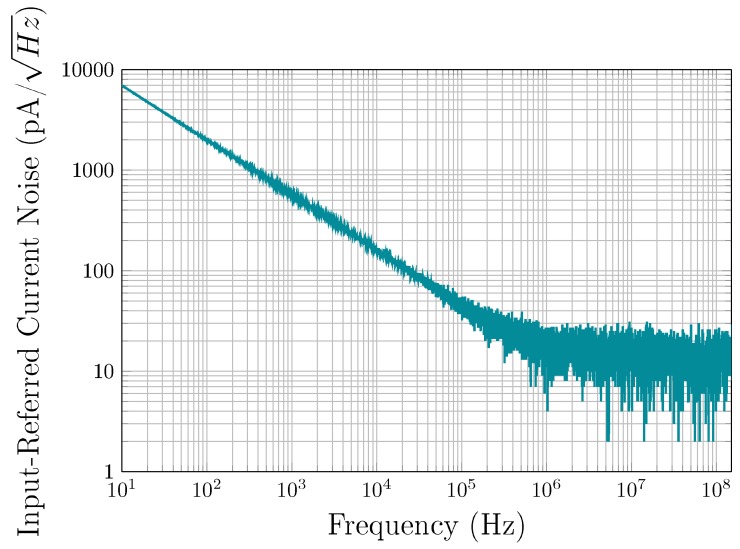
Measured TIA input-referred current noise.

**Figure 10:**
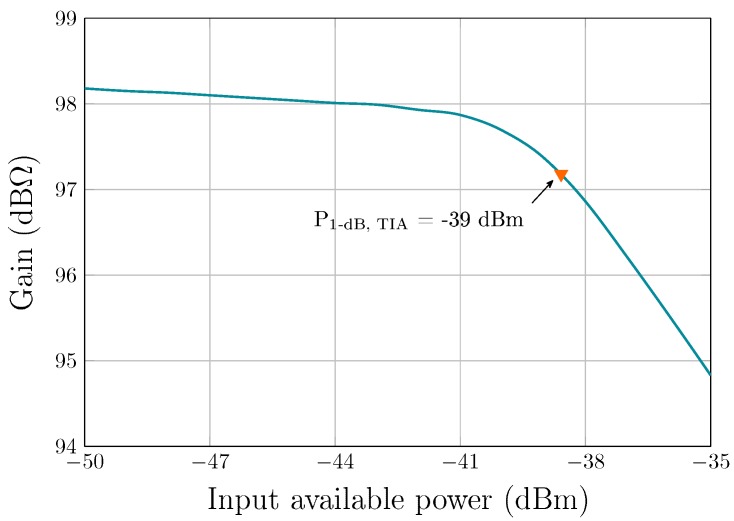
Measured TIA gain for different input power levels, outlining the 1-dB compression point.

The fully differential TIA is fabricated in a 65 nm CMOS process from TSMC, and consumes only 0.9 mA from a 1-V supply. The total circuit area measures 130 × 225 μm${}^{2}$, as shown in Figure 6. To obtain the frequency response of the TIA, S-parameters were measured using a Keysight E5061B VNA in a frequency range from 100 kHz to 1 GHz with an input power level of −45 dBm. Figure 8 shows the transimpedance gain and the 3-dB bandwidth of the TIA, versus two control signals, $V_{CTRL\_A}$ and $V_{CTRL\_BW}$. The maximum achievable gain is 98 dBΩ with a bandwidth of 90 MHz. The bandwidth can be extended to 142 MHz when the gain is reduced to 83 dBΩ. Control voltages can be varied independently in such a way that the gain and bandwidth are also independent from each other. As such, as $V_{CTRL\_BW}$ varies from 0.35 V to 0.45 V, the maximum gain variation (for the same $V_{CTRL\_A}$ value) is ∼0.32 dB (as seen in Figure 8a). The motional resistances of 35 kΩ and 8.8 kΩ, extracted from Figure 7 for $V_{p}$ of 100 V and 200 V, respectively, correspond to 91 dBΩ and 79 dBΩ, respectively, which can be covered by the maximum gain available of the proposed TIA to ensure sufficient gain for oscillation. Figure 9 shows the input-referred current noise of the TIA measured with a Keysight N9030A spectrum analyzer across a 142 MHz bandwidth. At low frequencies, the noise is dominated by the flicker noise, while the input current noise spectrum is flat in the frequency range from ∼500 kHz to 142 MHz where the input-referred noise is dominated by the white noise and reaches 15 pA/$\sqrt{\mathrm{Hz}}$. Figure 10 shows the measured transimpedance gain for different input power levels varying from −50 dBm to −35 dBm. The TIA 1-dB compression point was extracted to be of −39 dBm. The performance parameters of the TIA are summarized in Table 1.

### 4.3. MEMS Oscillator Characterization

#### 4.3.1. Open-Loop Measurements

**Figure 11:**
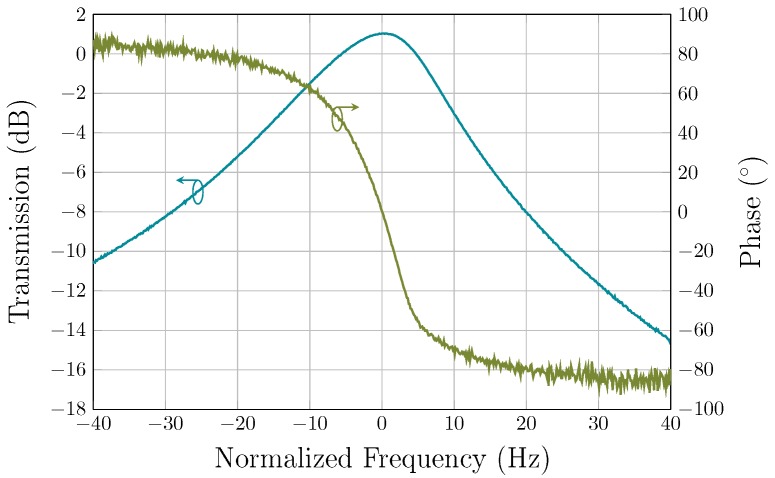
Measured open-loop gain and phase shift of the oscillator loop under vacuum at a polarization voltage of 100 V.

To confirm that sufficient loop gain was present for the oscillation, the resonator was connected to the TIA in open-loop configuration under vacuum, and the frequency and phase responses were measured using a Keysight E5061B VNA. As illustrated in Figure 6, the input and output ports of the VNA were connected to the differential inputs of the resonator and the differential outputs of the TIA, respectively, through external baluns which are used to convert between single-ended and differential signals. To sustain oscillation in closed-loop, the following conditions are required [37]:

(6) $$\phi_{total}=0^{{}^{\circ}},\;\mathrm{and}$$

(7) $$Z_{T}\geq R_{m}+R_{in}+R_{out},$$

where $\phi_{total}$ is the total phase shift, $R_{m}$ is the motional resistance of the resonator, and $Z_{T}$, $R_{in}$ and $R_{out}$ are the transimpedance gain, input, and output impedances of the TIA, respectively. In this case, both resonator and TIA must have 0° phase shift. The open-loop gain and phase characteristics are plotted in Figure 11. It is observed that the open-loop gain and phase shift at the resonant frequency of the resonator is higher than 0 dB and equal to 0°, respectively, as formulated in conditions (6) and (7), thus ensuring that oscillation can be sustained in closed-loop. In addition, the loaded Q-factor was measured from the open-loop gain bandwidth to be around 875,000.

#### 4.3.2. Closed-Loop Measurements

**Figure 12:**
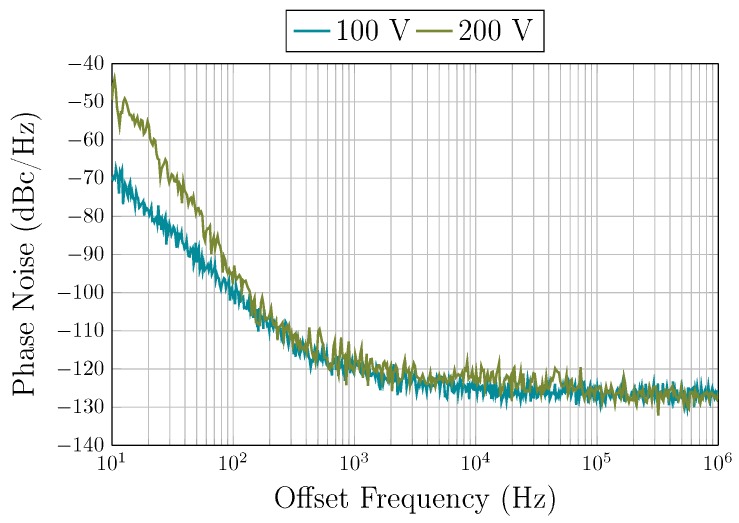
Measured phase noise in vacuum for polarization voltages of 100 V and 200 V.

**Figure 13:**
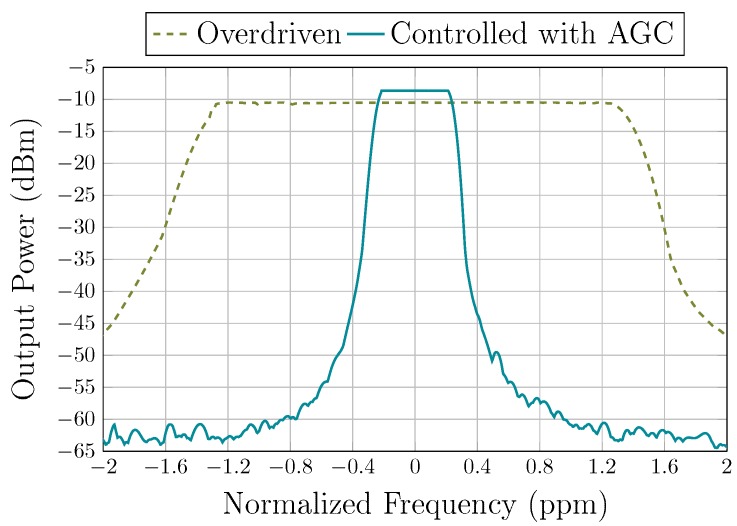
MEMS oscillator signal short time stability at a 17.93 MHz central frequency (averaged over a five-minute timespan) with a polarization voltage of 100 V.

The resonator and TIA were set in a closed-loop configuration (dashed lines in Figure 6) and tested under vacuum to characterize the performance of the oscillator. The expression for oscillator phase noise is given as follows [38]:

(8) $$L\left(f_{m}\right)\;=\;\frac{2FkT}{P_{0}}\times \left[1+{\left(\frac{f_{0}}{2Q_{L}\;f_{m}}\right)}^{2}\times \left(1+\frac{f_{c}}{f_{m}}\right)\right],$$

where *F* represents the noise factor of the amplifier, $P_{0}$ is defined as the oscillation power, $f_{m}$ the offset frequency from the carrier frequency, $f_{0}$ represents the carrier frequency, $f_{c}$ is a constant related to the 1/*f* noise corner of the oscillator and $Q_{L}$ denotes the loaded Q-factor and is defined as

(9) $$Q_{L}\;=\;Q_{UL}\times \frac{R_{m}}{R_{m}+R_{in}+R_{out}},$$

where $Q_{UL}$ is the intrinsic Q-factor of the resonator. The phase noise measurements of the oscillator under vacuum are plotted in Figure 12 for polarization voltages of 100 V and 200 V. The near-carrier phase noise at a 10 Hz offset was measured to be approximately of −50 dBc/Hz and of −70 dBc/Hz at polarization voltages of 100 V and 200 V, respectively. At an offset of 1 kHz, the phase noise was measured to be of −120 dBc/Hz at both polarization voltages. At a polarization voltage of 100 V, the TIA flicker noise dominates the close-to-carrier phase noise. However, at a polarization voltage of 200 V, the close-to-carrier phase noise is deteriorated by the resonator non-linearity [24]. Figure 7 clarifies the effect of the polarization voltage on the non-linearity, by comparing the response at 100 V and 200 V polarization. The resonator exhibits significant non-linearity at a −10 dBm signal input and 200 V polarization, whereas, the non-linearity is significantly reduced at 100 V polarization at the same signal level. This results in the phase noise in the close-to-carrier region to be improved by ∼20 dB when the polarization voltage is decreased. At farther frequency offsets, the phase noise reaches a floor of −127 dBc/Hz and is dominated by the TIA noise. These phase noise measurements translate in time-domain jitter values. The RMS integrated phase jitter (from 12 kHz to 20 MHz) is of 14 ps. Short-term stability is an important performance criterion of the oscillator and is a measure of its frequency stability. The frequency stability of the resonator is illustrated in Figure 13. The oscillator shows a broadening of the frequency output over a five-minute timespan. Please note that the frequency stability is improved when the AGC is used, from ±1.3 ppm to ±0.25 ppm, as this ensures that the non-linearity of the resonator is not exerted.

To allow for a representative comparison, two figure-of-merits FOM${}_{1}$, and FOM${}_{2}$, are used to evaluate the overall MEMS oscillator performance. Their expressions are respectively given by [19,39]

(10) $${\mathrm{FOM}}_{1}\;=\;L\left(f_{m}\right)-20\log\left(\frac{f_{0}}{f_{m}}\right)+10\log\left(\frac{P_{\mathrm{diss}}}{1\mathrm{mW}}\right),$$

and

(11) $${\mathrm{FOM}}_{2}\;=\;\frac{kT}{\mathrm{PN}\;\mathrm{Floor}\times P_{\mathrm{diss}}}f_{0}^{2}R_{m}^{2},$$

where $L(f_{m})$ is the oscillator phase noise at $f_{m}$, a specific offset frequency, $f_{0}$ is the center frequency, $P_{\mathrm{diss}}$ is the DC power consumption of the oscillator circuit (in mW), PN Floor is the phase noise floor (in dBc/Hz), and $R_{m}$ is the motional resistance. The calculated FOM${}_{1}$ and FOM${}_{2}$ values for different MEMS oscillators based on electrostatic resonators in the literature, both monolithic and non-monolithic, are listed in Table 2. It can be noticed that proposed FOM${}_{2}$ is used to evaluate the phase noise floor enabled by the TIA while considering the high resonator motional resistance [19]. The calculated FOM${}_{1}$ and FOM${}_{2}$ values for different MEMS oscillators based on electrostatic resonators in the literature are listed in Table 2. As can be seen, the MEMS-based oscillator demonstrated in this work has the highest figure-of-merit |FOM${}_{1}$| when compared to others [19,20,21,22,23,24,25,26], while [25,26] have higher FOM${}_{2}$, but exhibit significantly lower Q and higher motional resistance, ultimately leading to reduced phase noise performance. These works also present all-CMOS oscillators which impose more limitations on the material choices and resonator fabrication compared to the hybrid approach adopted in this work. Its close-to-carrier phase noise is notably lower because of the low noise of the TIA and the mitigation of the resonator non-linearity. Accordingly, the proposed design enables competitive performance while operating at a low power consumption of 0.9 mW.

Monolithic integration typically results in lower parasitic capacitances and consequently lower power dissipation, which is reflected in the FOMs, e.g., FOM${}_{1}$ for [21,24]. On the other hand, it imposes fabrication limitations on the resonator, e.g., thermal budget, materials, and processing steps compatibility with the electronics.

## 5. Conclusions

This paper presented a MEMS oscillator based on a Lamé-mode capacitive MEMS resonator and a fully differential high-gain TIA. The TIA was fabricated in a TSMC 65 nm CMOS process from TSMC and consumes 0.9 mW. An RGC input stage was used in this work to benefit from high gain, wide bandwidth, and lower input impedance which make it suitable for oscillators based on capacitive MEMS resonators. The TIA can reach a maximum gain of 98 dBΩ and has a bandwidth that is adjustable from 90 to 142 MHz, making it versatile for use with different resonators to attain suitable oscillation conditions. The input-referred current noise of the TIA was measured below 15 pA/$\sqrt{\mathrm{Hz}}$ in the mid-band. The proposed TIA was integrated with an 18-MHz Lamé-mode MEMS resonator to implement a MEMS oscillator. The presented MEMS oscillator achieves a phase noise of −120 dBc/Hz, at a 1-kHz offset and the phase noise floor is of −127 dBc/Hz. The oscillator exhibits a superior figure-of-merit relative to the state-of-the-art, notably in terms of power consumption and phase noise.

## Figures and Tables

**Table 1:** Performance parameters of the proposed TIA.

Parameter	Meas. Value
Transimpedance gain [dBΩ]	98
Bandwidth [MHz]	142
Input impedance, $R_{in}$ @$f_{0}$ [Ω]	89
Output impedance, $R_{out}$ $f_{0}$ [Ω]	100
Power supply, $V_{DD}$ [V]	1
Power Consumption, $P_{\mathrm{diss}}$ [mW]	0.9
1-dB compression point, $P_{1-\mathrm{dB}}$ [dBm]	−39
Input-referred noise @f${}_{0}$ $\left[\mathrm{pA}/\sqrt{\mathrm{Hz}}\right]$	14.5
Active area [mm${}^{2}$]	0.029
Process	65 nm CMOS

**Table 2:** Performance comparison of the proposed oscillator with the state-of-the-art.

	[19]	[20]	[21]	[22]	[23]	[24]	[25]	[26]	This Work
CMOS technology	0.35 μm	0.18 μm	0.35 μm	0.18 μm	0.35 μm	0.35 μm	0.35 μm	0.35 μm	65 nm
Gap [nm]	1500	200	100	50	80	60	450	900	2000
Center frequency, $f_{0}$ [MHz]	20	103	10.92	18	61.2	1.18	3.2	1.23	17.93
Testing condition	vacuum	air	vacuum	vacuum	air	vacuum	vacuum	vacuum	vacuum
Quality factor, Q	160,000	80,000	1092	8000	48,000	3029	2228	1900	889,539
Motional resistance, $R_{m}$ [kΩ]	65	5	6	76.9	15	700	12,000	16,000	35
Polarization voltage, $V_{p}$ [V]	26	18	5	2.5	12	45	30	7	100
AGC Circuit	Yes	Yes	No	No	No	No	No	No	Yes
Power supply, $V_{\mathrm{DD}}$ [V]	2.5	1.8	3.3	1.8	3.3	2.5	3.3	2.5	1
Power consumption, $P_{\mathrm{diss}}$ [mW]	6.9	2.6	0.35	5.9	0.95	1.3	1.21	0.15	0.9
PN @1kHz [dBc/Hz]	−105	−108	−80	−116	−100	−112	−82	−106	−120
PN Floor [dBc/Hz]	−131	−136	−96	−130	−130	−120	−105	−111	−127
FOM${}_{1}$ @1kHz [dB]	−183	−204	−165	−193	−196	−172	−71	−96	−205
FOM${}_{2}$ [Hz${}^{2}\Omega^{2}$]	3.6 × 10${}^{17}$	1.3 × 10${}^{19}$	1.9 × 10${}^{14}$	2.2 × 10${}^{18}$	1.7 × 10${}^{19}$	1.2 × 10${}^{19}$	1.58 × 10${}^{20}$	1.34 × 10${}^{21}$	3.8 × 10${}^{19}$
